# The Clinical Spectrum of Missense Mutations of the First Aspartic Acid of cbEGF-like Domains in Fibrillin-1 Including a Recessive Family

**DOI:** 10.1002/humu.21372

**Published:** 2010-12

**Authors:** Yvonne Hilhorst-Hofstee, Marry EB Rijlaarsdam, Arthur JHA Scholte, Marietta Swart-van den Berg, Michel IM Versteegh, Iris van der Schoot-van Velzen, Hans-Joachim Schäbitz, Emilia K Bijlsma, Marieke J Baars, Wilhelmina S Kerstjens-Frederikse, Jacques C Giltay, Ben C Hamel, Martijn H Breuning, Gerard Pals

**Affiliations:** 1 Department of Clinical Genetics, Leiden University Medical CenterLeiden, The Netherlands; 2 Department of Pediatric Cardiology, Leiden University Medical CenterLeiden, The Netherlands; 3 Department of Cardiology, Leiden University Medical CenterLeiden, The Netherlands; 4 Department of Ophthalmology, Leiden University Medical CenterLeiden, The Netherlands; 5 Department of Cardiothoracic Surgery, Leiden University Medical CenterLeiden, The Netherlands; 6 Bielefeld, Deutschland; 7 Department of Clinical Genetics, Academic Medical CenterAmsterdam, The Netherlands; 8 Department of Genetics, University Medical Center Groningen, University of GroningenGroningen, The Netherlands; 9 Department of Medical Genetics, University Medical Center UtrechtUtrecht, The Netherlands; 10 Department of Human Genetics, Radboud University Nijmegen Medical CenterNijmegen, The Netherlands; 11 Department of Clinical Genetics, Center for Connective Tissue Research, VU University Medical CenterAmsterdam, the Netherlands

**Keywords:** Marfan syndrome, fibrillin-1, *FBN1* gene, autosomal recessive inheritance, pathogenesis

## Abstract

Marfan syndrome (MFS) is a dominant disorder with a recognizable phenotype. In most patients with the classical phenotype mutations are found in the fibrillin-1 gene (*FBN1*) on chromosome 15q21. It is thought that most mutations act in a dominant negative way or through haploinsufficiency. In 9 index cases referred for MFS we detected heterozygous missense mutations in *FBN1* predicted to substitute the first aspartic acid of different calcium-binding Epidermal Growth Factor-like (cbEGF) fibrillin-1 domains. A similar mutation was found in homozygous state in 3 cases in a large consanguineous family. Heterozygous carriers of this mutation had no major skeletal, cardiovascular or ophthalmological features of MFS. In the literature 14 other heterozygous missense mutations are described leading to the substitution of the first aspartic acid of a cbEGF domain and resulting in a Marfan phenotype. Our data show that the phenotypic effect of aspartic acid substitutions in the first position of a cbEGF domain can range from asymptomatic to a severe neonatal phenotype. The recessive nature with reduced expression of *FBN1* in one of the families suggests a threshold model combined with a mild functional defect of this specific mutation. © 2010 Wiley-Liss, Inc.

## INTRODUCTION

Human fibrillin-1 is a large protein of approximately 350 kD and member of a family of extracellular cysteine-rich glycoproteins. Since 1991 mutations in the fibrillin-1 *(FBN1)* gene have been found to be responsible for Marfan syndrome (MFS; MIM# 134797) (Kainulainen et al., 1990; Dietz et al., 1991). Fibrillin-1 is characterized by a highly conserved modular domain organization. The most prominent domain is the Epidermal Growth Factor-like (EGF) domain present 46 times and containing six highly conserved cysteine residues stabilizing the structure by three disulfide bonds. Of these EGF domains, 43 have a consensus sequence for calcium binding (cb) in the N-terminal pocket of the domain which may mediate protein-protein interactions. The EGF domains are interrupted by seven transforming growth factor (TGF)-binding protein domains characterized by 8 cysteine residues involved in intra-domain disulfide bonds (Pereira et al., 1993; Robinson et al., 2006). In the last update of the Universal Marfan Database - *FBN1 (UMD-FBN;* http://www.umd.be) (Faivre et al., 2007) 803 different mutations are reported. Most of the mutations are missense mutations (56%) mainly substituting or creating a cysteine in a cbEGF domain. Other mutations are frameshift mutations, splice mutations and nonsense mutations. About 14% of mutations are recurring.

All cbEGF domains start with a highly conserved aspartic acid, which is crucial for binding of a positively charged Ca^2+^ ion (Whiteman et al., 2007). We have identified 10 index cases with a substitution of the first aspartic acid substitution of a cbEGF domain and reviewed a further 14 published cases. Most of them exhibit a complete MFS phenotype. Surprisingly, in one family the substitution only led to MFS in homozygous state in three family members, whereas 13 family members carrying the heterozygous mutation do not have Marfan syndrome after thorough clinical examination.

There is still a lot of debate how mutations in *FBN1* result in the MFS phenotype, but increasing evidence for different models is emerging. Possible explanations for the observed extreme variation in expression of the substitution of the first aspartic acid of a cbEGF domain are discussed. The observation of recessive inheritance of an expected dominant mutation also underscores the fact that mutations which are predicted to have a pathogenic effect, may not always lead to clinical symptoms.

## PATIENTS AND METHODS

### Patients

The patients were referred for DNA analysis of the fibrillin-1 gene to confirm the clinical diagnosis of MFS. Case 1, 2, 5, 7, 9 and 10 fulfilled the clinical Ghent criteria (De Paepe A. et al., 1996) for the diagnosis MFS. All index patients fulfilled the Ghent criteria when the finding of a pathogenic mutation in *FBN1* was included.

**Figure 1:**
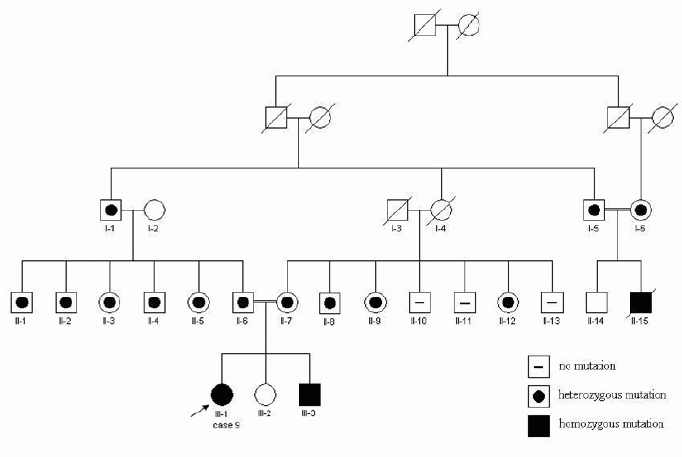
Pedigree of the family of case 9. Squares, male subjects; circles, female subjects. Affected subjects with a homozygous mutation (c.7454A>T) are represented by solid symbols. Presence or absence of the heterozygous mutation is represented by an open symbol with a black dot or a minus symbol respectively.

Case 9 belongs to a large Turkish pedigree (Figure 1, III-1). She was examined at the age of 22 years. At the age of 6 weeks she was operated on a right sided hernia inguinalis. She was diagnosed with bilateral subluxation of the lenses when she was 3 years old. From that time on she has been operated several times for retinal detachments and lens luxation. At the age of 14 years an aortic root replacement was performed for progressive aortic root dilatation and aortic valve regurgitation. A spontaneous pneumothorax occurred at the age of 16 years.

Clinical examination at the age of 18 years showed a marfanoid habitus, slight downslanting of palpebral fissures and a high and gothic palate. Despite long fingers, wrist and thumb signs were negative. She exhibited limited extension of her elbows, mild asymmetry of the chest, and bilateral flat feet. Her skin showed several striae on the chest, shoulders, hips and lower back. Her length was 179.5 cm (+3,7SD for Turkish descent) and an arm span of 175 cm (within normal limits). The brother of case 9 (III-3) was a 13 year old boy with Marfan syndrome. He had mild skeletal manifestations of Marfan (pes planus), a mildly dilated aortic root, ectopia lentis and dural ectasia with an anterior sacral meningocele. Furthermore he suffered from recurrent episodes of intracranial hypertension treated by drainage of cerebrospinal fluid. He has been described in a case report (Hilhorst-Hofstee et al., 2008).

The third patient (II-15) died at the age of 22 years. His case history was obtained from the medical records. At the age of 2 years bilateral subluxation of lenses was diagnosed. He developed severe aortic and mitral valve regurgitation with an aneurysm of the aortic root. He had skeletal involvement and an anterior sacral meningocele. When he was 17 years of age an aortic root replacement was performed with reconstruction of the aortic and mitral valve. Due to progressive aortic regurgitation, a re-operation was performed a year later. He died at the age of 22 after a second episode of ventricular fibrillation.

All heterozygous family members had a thorough skeletal, cardiologic and ophthalmologic examination including anthropometric measurements, echocardiography and slit lamp evaluation (Table 1). Only the mother and father of case 9 had an MRI evaluation for dural ectasia. The obtained clinical data of all family members are summarized in Table 1. The father of case 9 (II-6) had no clinical signs of Marfan syndrome. The mother (II-7) was tall, with a height on +2.5 SD but with normal body proportions. She had no other skeletal, ocular or cardiovascular involvement, but had several striae on the lumbar region and around the knees. Furthermore she suffered from spontaneous pneumothorax at the age of 21 years. An MRI-scan showed a dural ectasia at S2 with otherwise a normal dural sac. The father and mother of II-15 did not exhibit any signs of Marfan syndrome. None of the nine other heterozygous family members had a major criterion in one of the organ systems. Some were found to have a non-specific or minor sign. Individual II-1 has an arm span to height ratio of 1.06 and a mild dilatation of the abdominal aorta. II-4 had recurrent inguinal hernias but this was during a period of performing heavy physical labor. II-8 had an arm span to height ratio of 1.07 and bilateral flat feet. II-9 had reduced extension of the elbows.

**Table 1:** Summary of the clinical features in the family of case 9

	**III-1**															
	**Case 9**	**III-3**	**II-6**	**II-7**	**II-15**	**I-5**	**I-6**	**I-1**	**II-1**	**II-2**	**II-3**	**II-4**	**II-5**	**II-8**	**II-9**	**II-12**
Age at examination (years)	10	22	42	43	9	57	56	61	44	41	37	35	33	55	48	37
Sex	M	F	M	F	M	M	F	M	M	M	F	M	F	M	F	F
height (cm)	150,5	180,9	167,1	174,4	135,0	164,0	157,5	166,0	167,5	174,5	167,0	188,5	161,0	181,0	164,7	161,0
height SDS (for Turkish descent)	1,6	3,7	-1,2	2,5	-0,2	-1,7	-0,6	-1,4	-1,1	0,1	1,1	2,5	0,8	1,2	0,7	0,2
arm span : height ratio	1,02	0,97	1,04	1,00	1,00	1,01	1,03	1,01	1,06	1,04	0,98	0,99	1,02	1,07	1,02	1,03
sitting height: height ratio	0,488	0,515	0,536	0,513	np	0,535	0,528	0,519	0,527	0,517	0,531	0,516	0,523	0,482	0,520	0,526
sitting height: height ratio SDS	-2,0	-0,8	1,6	-0,8	np	1,5	0,2	0,3	1,0	0,3	0,3	0,2	0,0	-1,8	-0,4	0,0
**Skeletal system**	**involv**	**involv**	**none**	**none**	**involv**	**none**	**none**	**none**	**none**	**none**	**none**	**none**	**none**	**involv**	**none**	**none**
**major**																
pectus carinatum	no	no	no	no	yes	no	no	no	no	no	no	no	no	no	no	no
pectus excavatum requiring	no	no	no	no	no	no	no	no	no	no	no	no	no	no	no	no
surgery																
sitting height: height ratio <2																
SD or armspan : height ratio	yes	no	no	no	np	no	no	no	yes	no	no	no	no	yes	no	no
>1.05																
wrist and thumbsigns	no	no	no	no	no	no	no	no	no	no	no	no	no	no	no	no
scoliosis of >20° or																
spondylolisthesis	no	no	no	no	np	no	no	no	no	no	no	no	no	no	no	no
reduced extension at the elbow																
(<170°)	no	yes	no	yes	np	no	no	no	no	no	no	no	no	no	yes	no
pes planus	yes	yes	no	no	yes	no	no	no	no	no	no	yes	no	yes	no	no
protrusio acetabulae	np	np	np	np	np	np	np	np	np	np	np	np	np	np	np	np
**minor**																
pectus excavatum of moderate severity	no	no	no	no	yes	no	no	no	no	no	no	no	no	no	no	no
joint hypermobility	no	no	no	no	np	no	no	no	no	no	no	no	no	no	no	no
highly arched palate with crowding	no	yes	no	no	yes	no	no	no	no	no	no	no	no	no	no	no
facial appearance	no	yes	no	no	yes	no	no	no	no	no	no	no	no	no	no	no
**Ocular system**	**major**	**major**	**none**	**none**	**major**	**none**	**none**	**none**	**none**	**none**	**none**	**none**	**none**	**none**	**none**	**none**
**major**																
ectopia lentis	yes	yes	no	no	yes	no	no	no	no	no	no	no	no	no	no	no
**minor**																
abnormally flat cornea	np	np	np	np	np	no	no	no	no	no	no	no	no	no	no	no
increased axial length of globe	np	np	np	np	np	np	np	np	np	np	np	np	no	no	no	no
hypoplastic iris or ciliary muscle	np	np	no	no	yes	no	no	no	no	no	no	no	no	no	no	no
**Cardiovascular system**	**major**	**major**	**none**	**none**	**major**	**none**	**none**	**none**	**involv**	**none**	**none**	**none**	**none**	**none**	**none**	**none**
**major**																
Z-score aortic root diameter	10,5^1^	**4**	**0.3**	**-1.4**	**>2^2^**	**-0.9**	**-0.8**	**-1.8**	**1.4**	**-1.1**	**-0.8**	**-2.9**	**-2.5**	**0.4**	**0.5**	**0.8**
dilatation ascending aorta	yes (arr)	yes	no	no	yes (arr)	no	no	no	no	no	no	no	no	no	no	no
dissection of ascending aorta	no	no	no	no	no	no	no	no	no	no	no	no	no	no	no	no
**minor**																
mitral valve prolaps	yes	yes	no	no	yes	no	no	no	no	no	no	no	no	no	no	no
dilatation of main pulmonary artery	no	no	no	no	np	no	no	no	no	no	no	no	no	no	no	no
calcification of the mitral annulus < 40 years	no	no	no	no	np	no	no	np	no	no	no	no	no	no	no	no
dilatation or dissection of descending aorta < 50 years	no	no	np	no	np	no	no	no	yes	no	no	no	no	no	no	no
**Pulmonary system**	**involv**	**none**	**none**	**involv**	**none**	**none**	**none**	**none**	**none**	**none**	**none**	**none**	**none**	**none**	**none**	**none**
**minor**																
spontanous pneumothorax or apical blebs	yes	no	no	yes	no	no	no	no	no	no	no	no	no	no	no	no
**Skin and integument**	**involv**	**none**	**none**	**involv**	**np**	**none**	**none**	**none**	**none**	**none**	**involv**	**involv**	**none**	**none**	**none**	**none**
**minor**																
striae atrophicae recurrent or incisional herniae	yes no	no no	no no	yes no	np np	no no	no no	no no	no no	no no	yes no	no yes^3^	no no	no no	no no	no no
**Dura**	**np**	**major**	**none**	**major**	**np**	**np**	**np**	**np**	**np**	**np**	**np**	**np**	**np**	**np**	**np**	**np**
**major**																
lumbosacral dural ectasia	np	yes	no	yes	np	np	np	np	np	np	np	np	np	np	np	np
**Family or genetic history**	**major^4^**	**major^4^**	**major^4^**	**major^4^**	**major^4^**	**major^4^**	**major^4^**	**major^4^**	**major^4^**	**major^4^**	**major^4^**	**major^4^**	**major^4^**	**major^4^**	**major^4^**	**major**
**major**																
1st degree relative with Marfan syndrome pathogenic mutation in *FBN1*	no hom	yes hom	yes het	yes het	no hom	yes het	yes het	no het	no het	no het	no het	no het	no het	no het	no het	no het

F female; M male; involve involvement; np not performed; arr aortic root replacement; homh mutation; Z-score related to body surface area and age according to Roman et al. (Roman et al., 1989); 1) aortic root measurement at the age of 14 years just before aortic root replacement; replacement; 2) no exact measurement available; 3) recurrent inguinal hernias during a period member or the presence of a pathogenic *FBN1* mutation.

The clinical data of cases 1-10 and published cases are summarized in Table 2 together with the molecular data.

**Table 2:** Published and observed missense mutations leading to the substitution of the first aspartic acid of a cbEGF domain

**Nucleotide change**	**Amino acid change**	**Exon**	**cbEGF domain**	**Diagnosis**	**Phenotype**	**Reference**	**Aberant mRNA splicing**	**Predicted aberant mRNA splicing^a^**	**Inheritance**
C.1468OT	p.Asp490Tyr	**11**	**#3**	Classical MFS	ard, el, sk	(Hayward and Brock, 1997)	np	yes	Unknown
c.2168A>C	p.Asp723Ala	**18**	**#7**	Severe classical MFS	ard, mvp, el, myop, sk	(Dietz etal., 1993)	np	no	De novo
c.2168A>T	p.Asp723Val	**18**	**#7**	Classical MFS	ard, mvp, el, sk, myop	(Katzke etal., 2002)	np	no	De novo
c.2728G>A	p.Asp910Asn	**22**	**#10**	Classical MFS	unknown	UMD	np	yes	Unknown
c.3209A>G	p.Asp1070Gly	**26**	**#12**	Neonatal MFS	unknown	UMD	np	no	Unknown
c.3338A>G	p.Asp1113Gly	**27**	**#13**	Phenotype unknown	unknown	(Liu etal., 1997)	np	no	Unknown
c.3463G>A	p.Asp1155Asn	**27**	**#14**	Thoracic aortic aneurysm	ard, mvp, diss	(Milewicz et al., 1996)	no	yes	De novo
c.3464A>G	p.Asp1155Gly	**28**	**#14**	Classical MFS	ard, el	**Case** 1	no	no	Parents not available, 5 sibs neg for mutation
c.3712G>A	p.Asp1238Asn	**29**	**#16**	Phenotype unknown	unknown	(Yuan etal., 1999)	np	no	Unknown
c.3713A>G	p.Asp1238Gly	**30**	**#16**	Classical MFS	ard, mvp, sk	(Tiecke etal., 2001)	np	no	Familial
c.3964G>A	p.Asp1322Asn	**31**	**#18**	Neonatal MFS	ard, mi, ti, myop, sk	**Case** 2	np	yes	De novo
c.4210G>T	p.Asp1 404Tyr	**33**	**#20**	Classical MFS	ard, el, sk	(Hayward et al., 1997)	yes	yes	Familial
c.5422G>C	p.Asp1808His	**43**	**#26**	Lens luxation and striae	el, str	**Case** 3	np	no	Parents not available
c.5671G>A	p.Asp1891His	**45**	**#28**	Classical MFS	ard, sk	**Case** 4	np	no	De novo
c.5788G>C	p.Asp1930His	**45**	**#29**	Classical MFS	ard, el, sk	**Case** 5	np	yes	De novo
c.5788G>A	p.Asp1930Asn	**46**	**#29**	Phenotype unknown	unknown	(Liu etal., 1997)	np	yes	Unknown
c.5788G>A	p.Asp1930Asn	**46**	**#29**	Classical MFS	ard, el, sk, de, str	**Case** 6	np	yes	Parents not available
c.6037G>T	p.Asp2013 Tyr	**48**	**#31**	Classical MFS	ard, el, sk, de, str	**Case** 7	np	yes	Familial
c.6379G>T	p.Asp2127Tyr	**51**	**#32**	Classical MFS	ard, el	(Matsukawa et al., 2001)	np	yes	Familial
c.6381T>A	p.Asp2127Glu	**52**	**#32**	Classical MFS	ard, sk	(Kainulainen etal., 1994)	np	no	Familial
c.7331A>G	p.Asp2444Gly	**59**	**#38**	Classical MFS	ard, sk	**Case** 8	np	no	De novo
c.7454A>T	p.Asp2485Val	**60**	**#39**	Classical MFS in homozygous state	ard, el, sk, str, her, pn	**Case** 9		no	Familial
c.7819G>A	p.Asp2607Asn	**62**	**#42**	Classical MFS	ard, mvp, sk	**Case** 10	no	no	Mother suspect for MFS
c.7820A>G	p.Asp2607Gly	**63**	**#42**	Phenotype unknown	unkown	(Liu etal., 1997)	np	no	Unknown

UMD Universal Marfan Database - *FBN1 (UMD-FBN;* http://www.umd.be); cbEGF calcium binding Epidermal Growth Factor domain; bp basepair; np not performed; neg negative; pos positive; ard aortic root dilatation; diss aortic dissection; mvp mitral valve prolapse, mi mitral valve insufficiency; el ectopia lentis; pal high arched palate; ti tricuspid valve insufficiency; myop high myopia; ar arachnodactyly; hm hypermobility; contr contractures; str striae; her hernia; sk skeletal involvement; de dural ectasia; pn pneumothorax; unknown. The gray row represents the recessive mutation described in this article.

Mutation numbering refers to the *FBN1* cDNA GenBank reference sequence: NM_000138.3, with the A of the ATG translation initiation codon as nucleotide +1 (http://www.hgvs.org/mutnomen).^a^) Alamut mutation interpretation software version 1.5; Interactive Biosoftware, Rouen France.

### Molecular studies

DNA was extracted from peripheral blood or paraffin embedded tissue, using standard techniques, analyzed by DHPLC and direct DNA sequencing as described previously (Matyas et al., 2002).

The reference sequence used to describe the mutations is the *FBN1* cDNA GenBank reference sequence: NM_000138.3. Nucleotide numbering reflects cDNA numbering with +1 corresponding to the A of the ATG translation initiation codon in the reference sequence, according to journal guidelines (http://www.hgvs.org/mutnomen).

Skin biopsies of case 1, 9 and her mother and case 10 were available. Fibroblasts were cultured and mRNA was isolated from confluent monolayers. For each individual two RNA isolations were performed: one cell culture of each was incubated with cycloheximide (0.25 mg/ml) for 4.5 hours prior to RNA isolation, to prevent possible nonsense mediated decay (NMD) of aberrantly spliced mRNA.

RNA was isolated using the RNA isolation minikit (Qiagen) according to the manufacturers instructions. Full length single stranded cDNA was prepared with oligo-dT-primer and Superscript^T^MII RT reverse trancriptase (Invitrogen). To detect possible splice errors, the complete coding sequence of *FBN1* was analyzed by direct sequencing in 24 overlapping PCR fragments. The primers for overlapping fragments were positioned in different exons, to avoid allele dropout in case of exon skipping. For analysis of the mutation in exon 60, primers in exon 55 (forward) and 63 (reverse) were used.

Primer sequences are given in Supp. Table S1.

## RESULTS

Heterozygous mutations leading to a substitution of the first aspartic acid of a cbEGF domain were found in nine index cases and a homozygous mutation was found in 1 index case. These mutations and 14 comparable mutations described in the literature or in the *UMD-FBN1 (UMD-FBN;* http://www.umd.be) are listed in Table 2. The phenotypes were classical MFS in 15 cases, neonatal MFS in two cases, thoracic aortic aneurysm in one case and lens luxation with striae in one case. In four cases described in the literature the phenotype is not clear.

The mutation was de novo in cases 2, 4, 5, and 8. In case 1, 3 and 6 parental DNA was not available. In case 1 five sibs were tested negative for the mutation. In case 6 the parents and seven sibs had no clinical symptoms of MFS. Case 7 has an affected sister with the same mutation. Their father died as a consequence of his third aortic dissection when he was 52 years of age. He was thought to have MFS. The mother of case 10 is probably affected but was not molecularly tested. Of the published mutations three were de novo, three were familial and in eight inheritance was unknown (Table 2).

Using DHPLC for mutation scanning of all 65 exons of *FBN1,* initially no mutation was found in case 9. As the parents are consanguineous, a recessive mutation was suspected. Testing one of the parents, a heterozygous mutation was detected in exon 60: c.7454A>T, leading to the amino acid substitution p.Asp2485Val. This mutation was found in a homozygous state in the patients III-1, III-3 and II-15. The parents (II-6 and II-7) of patients III-1 and III-3 are first cousins. The parents of patient II-15 are distantly related and both are related to II-6 and II-7 (Figure 1). In the parents of patients III-1, III-3 and II-15 and 9 family members the mutation was heterozygous. The mutation was not detected in 1000 Caucasian and 60 Turkish control chromosomes.

Sequence analysis of cDNA, made from mRNA from cultured fibroblasts of case 1, case 9 and her mother, and case 10 showed no evidence of erroneous splicing of exon 60. Treatment with cycloheximide, to prevent possible nonsense mediated decay of erroneously spliced mRNA, gave the same results. Fibrobasts of the other patients were not available.

## DISCUSSION

We identified a heterozygous substitution of the first aspartic acid of a cbEGF domain in *FBN1* in nine index patients and a homozygous substitution in one index patient with MFS. Reviewing the literature we found 12 reports of substitution of the first aspartic acid, and a further two unpublished cases are quoted in the UMD database *(UMD-FBN;* http://www.umd.be) (Collod-Beroud et al., 2003) as summarized in Table 2. All 10 index patients found in our center fulfilled the Ghent criteria when the finding of a pathogenic mutation was included. Of the 14 published cases, eight were reported to have a classical Marfan phenotype, one was a neonatal Marfan and one had a thoracic aortic aneurysm. Of four cases the phenotype was not reported. In all cases the acidic amino acid aspartic acid is replaced by a nonpolar or noncharged polar amino acid, apart from one mutation where aspartic acid is replaced by another acidic amino acid (p.Asp2127Glu) (Kainulainen et al., 1994). The codons of the first aspartic acids in the cbEGF domains always contain the last base of one exon and the first two bases of the next. Consequently, mutations of these codons may affect splicing. Aberrant splicing was excluded in cases 1, 9, and 10 and in one of the published cases (Milewicz et al., 1996). The mutation c.4210G>T was shown to destroy a donor splice site with abnormal splicing as a consequence (Hayward et al., 1997). Of the 19 cases in which no cDNA analysis was performed, prediction software predicted aberrant splicing in eight cases (Table 2). Exon skipping, as a result of these mutations, may have more severe effects than missense mutations, because the exons are all in frame and consequently, skipping will lead to a shorter protein that may exert a dominant negative effect (Robinson etal., 2002).

**Figure 2:**
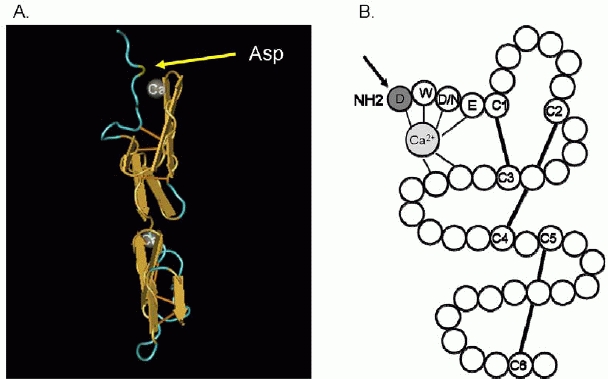
Class I cbEGF domain showing the position of the first Asp in relation to the calcium molecule. (A) 3D picture of a cbEGF domain of fibrillin-1. The yellow arrow points to the first Asp. Picture derived from the NCBI database (http://www.ncbi.nlm.nih.gov/) (Downing et al., 1996). (B) cbEGD like domain (Handford et al., 1991). The arrow points to the first aspartic acid residue. Solid lines are the disulphide bridges between cysteine residues.

There are several reasons to argue that a substitution of the first aspartic acid of a cbEGF domain will lead to a MFS phenotype in the heterozygous state. Calcium binding of cbEGF domains is necessary for stabilization of the secondary structure, prevention of proteolytic degradation and for protein-protein interaction (Dietz et al., 1993; Handford et al., 1991; Cooke et al., 1987). The first aspartic acid of a cbEGF domain is highly conserved in evolution and in the human fibrillin-1 gene all cbEGF domains start with an aspartic acid, which is crucial for binding of a positively charged Ca^2+^ ion (Figure 2) (Whiteman et al., 2007). Furthermore mutations of the first amino acid of a cbEGF domain of coagulation factor IX in haemophilia B have been proven to reduce calcium binding even if the aspartic acid is replaced by the acidic amino acid glutamic acid (Handford et al., 1991; Winship and Dragon, 1991). The finding of 23 MFS or MFS-like cases with a heterozygous substitution of an aspartic acid in this position of the cbEGF domain underscores the crucial role of this amino acid. In this view the recessive nature of the mutation p.Asp2485Val in the family of case 9 came as a surprise. The family of case 9 (Figure 1) has been thoroughly investigated. Patients III-1 (case 9), III-3 and II-15 have the classical type of Marfan syndrome according to the Ghent criteria (De Paepe A. et al., 1996). Based on the pedigree with three affected patients and healthy consanguineous parents recessive inheritance could be expected. This was confirmed by finding a homozygous missense mutation in all three affected patients. The four unaffected parents and nine other unaffected relatives were found to be carriers of the mutation. Unexpectedly in none of the investigated heterozygous carriers obvious signs of Marfan syndrome could be found. Only after thorough clinical examination one of them (II-7) was found to have a dural ectasia at S2, which as yet is considered a major symptom in the Ghent criteria. Together with the family history and some minor signs (pneumothorax, striae and reduced extension of the elbows) this classifies II-7 as having Marfan syndrome. However, compared to the homozygous cases, the cardinal Marfan features in the skeletal, cardiac and ophthalmological systems are absent.

To our knowledge very little is known about recessive *FBN1* mutations. Only one family has been reported in which Marfan syndrome was found in two affected cousins homozygous for a *FBN1* mutation while the four normal parents were heterozygous carriers, indicating recessive inheritance of the syndrome (De Vries et al., 2007). The mutation is located in exon 11 and leads to an amino-acid substitution creating a cysteine. Like us the authors expected this mutation to have a dominant effect in the heterozygous state. Two of the parents were sibs and exhibited minor signs of Marfan syndrome (increased arm span to height ratio and a highly arched palate). No other family members have been investigated. Only one other probably recessively inherited form of Marfan syndrome has been described but could not be proven by molecular analysis as the gene was not yet known (Fried and Krakowsky, 1977).

Three families have been described in the literature in which both parents are affected with more severely affected children. The first is an Italian couple of first cousins, both affected with Marfan syndrome, who had 4 affected children. Two of the four affected children showed more severe manifestations than other affected family members, presumably due to homozygosity (Capotorti L. et al., 1959). In 1984, Chemke described a family with Marfan syndrome. Two sibs suffered from a severe phenotype reminiscent of neonatal Marfan syndrome. Their parents were cousins and had a much milder phenotype. Remarkable is that the probably homozygous sibs were the only patients in the family with ectopia lentis (Chemke et al., 1984). In 1988 a severely affected boy has been described (Schollin et al., 1988). Both parents were affected and were found to carry amissense mutation in *FBN1.* Compound heterozygosity was identified in the severely affected child (Karttunen et al., 1994).

In the recessive family described here the heterozygous mutation does not exert an important effect on the phenotype. Only in the homozygous state the abnormal fibrillin causes the classical clinical phenotype of Marfan syndrome. This observation together with the few other described families with bi-allelic inheritance, may support both alternative pathogenetic models of Marfan syndrome. A dominant negative effect of *FBN1* mutations has been the leading hypothesis for the pathogenesis of Marfan syndrome for a long time. However, several manifestations of Marfan syndrome like bone overgrowth, craniofacial features, lung disease, and muscle and fat hypoplasia could not be explained by a structurally abnormal protein. The observation that fibrillin interacts with a variety of proteins, including the latent TGFβ binding proteins (LTBP's) has lead to several investigations indicating that fibrillin-1 can interact with TGF^β^ signaling (Annes et al., 2003; Azhar et al., 2003; Isogai et al., 2003; Hubmacher et al., 2006; Kaartinen and Warburton, 2003; Loeys et al., 2006; Neptune et al., 2003; Ng et al., 2004; Ten Dijke and Arthur, 2007).

According to splice site prediction software (Alamut mutation interpretation software version 1.5; Interactive Biosoftware, Rouen, France) the c.7454A>T mutation, found in the family described here, is not predicted to cause erroneous splicing. This was confirmed by cDNA studies, showing no evidence of splice error. The position of the mutation (exon 60) may explain the lack of expression as mutations in the more C-terminal end of the gene are expected to give a milder phenotype (Faivre et al., 2007; Robinson et al., 2002). However the mutation in case 10 is even more terminal and still leads to a classical MFS phenotype.

Hutchinson et al. (Hutchinson et al., 2003) hypothesized that variable expression of the normal *FBN1* allele could moderate the phenotypic effect of the mutant allele. A compensatory higher level of *FBN1* expression from the normal allele would explain a milder phenotype. As the normal alleles are inherited from 5 different parents in our recessive family, this mechanism is highly unlikely.

We hypothesize that the p.Asp2485Val mutation acts as a hypomorphic allele with a minimal dominant negative effect. Reduction of gene expression of both alleles could be the main determinant of the phenotype in homozygotes. The observation of only one major clinical sign in one of the heterozygotes (dural ectasia in II-7, Figure 1) and no major clinical signs in 12 other heterozygotes could be explained by sufficient gene expression with only a mild functional defect of the mutant allele product. This was also shown in a mouse model, however in this model the mutation had a severe dominant negative effect (Pereira et al., 1997). In this model with a deletion of 272 amino acids in the central part of fibrillin-1, a tenfold reduction in expression of the mutant allele was shown in heterozygous mice, resulting in a normal phenotype. Homozygous mutant mice however died shortly after birth due to severe vascular complications. The other mouse model of Pereira was a targeted *FBN1* mutation leading to 15% expression of a normal product with no abnormal phenotype in heterozygous mice, while mice homozygous for this mutation have severe abnormalities comparable with the neonatal MFS phenotype. These mouse models suggests that there is a threshold of expression of the normal allele below which the abnormal phenotype will develop (Pereira et al., 1999; Dietz and Mecham, 2000).

To understand the exact pathogenetic mechanism expression studies and studies on protein synthesis, secretion and matrix incorporation of the Asp2486Val mutation are necessary For comprehensive studies of this type, a mouse model should be created.

The finding of a homozygous substitution of A>T, as described here, has implications for mutation screening in MFS. Homozygous substitutions will not have an effect on denaturation of double stranded DNA, because the basepair remains the same. Consequently, heteroduplex based testing, such as DHPLC, working with the principle of differential denaturation of double stranded heteroduplex DNA, cannot detect this mutation in homozygous state. Formerly, based on the presumed dominant inheritance mode, only heterozygous mutations were expected. Now it is clear that recessive inheritance is also possible and mutations may have been missed in similar cases, because heteroduplex based testing has been used until recently in many large diagnostic centers. Most laboratories nowadays use direct sequencing, which avoids this problem.

In conclusion we have shown that the first aspartic acid of a cbEGF domain in *FBN1* is important for the function of fibrillin-1, but may not always lead to a clinical effect in the heterozygous state. This underscores that missense mutations must be interpreted with care.

## Figures and Tables

**Supp. Table S1:** Primers for cDNA sequencing of *FBN1:* F=forward; R=reverse; numbers refer to position in cDNA sequence

fragment	primername	Sequence 5’ > 3’	Length bp
1	FBN1F1	ATGCGTCGAGGGCGTCTGCT	384
	FBN1R384	GCTACCTCCATTCATACAGCGA	
2	FBN1F318	GATAGCTCCTTCCTGTGGCTCC	406
	FBN1R728	CCGTGCGGATATTTGGAATG	
3	FBN1F657	CCCCTGTGAGATGTGTCCTG	407
	FBN1R1064	TTGGTTATGGACTGTGGCAGC	
4	FBN1F1012	ACAGCTCTGACAAACGGGCG	384
	FBN1R1396	TGCAGCGTCCATTTTGACAG	
5	FBN1F1358	GCCAGTTGGTCCGCTATCTC	330
	FBN1R1688	ACATGAAAGCCCGCATTACAC	
6	FBN1F1609	AATGGCCGGATCTGCAATAA	398
	FBN1R2007	CTGGCCTCTCTTGTATCCACCA	
7	FBN1F1927	CTGGCTGTGGGTCTGGATGG	362
	FBN1R2289	GCAGTTTTTCCCAGTTGAATCC	
8	FBN1F2212	ATCTGTGAAAACCTTCGTGGGA	399
	FBN1R2611	AGGTGGCTCCATTGATGTTGA	
9	FBN1F2458	GTCTGCAAGAACAGCCCAGG	357
	FBN1R2815	TGGGACACTGACACTTGAATGA	
10	FBN1F2699	TACTCAAGAATTAAAGGAACA	525
	FBN1R3224	CGGCATTCGTCAATGTCTGTGC	
11	FBN1F3141	CATTGGCAGCTTTAAGTGCAGG	460
	FBN1R3621	ACCACCATTCATTATGCTGCA	
12	FBN1F3558	CCATTCAACTCCCGATAGGCT	335
	FBN1R3893	TTTTCACAGGTCCCACTTAGGC	
13	FBN1F3783	CAGGTGCTTGTGTTATGATG	392
	FBN1R4173	GCACAGACAGCGGTAAGA	
14	FBN1F4062	GATTGGAGATGGCATTAAGTGC	451
	FBN1R4513	TGTTGACACAGTTCCCACTGA	
15	FBN1F4425	CTACGAACTGGACAGAAGCGG	593
	FBN1R5018	ATACAGGTGTAGTTGCCAACGG	
16	FBN1F4910	ACTACCTGAATGAAGATACACG	626
	FBN1R5536	GACCTGTGGAGGTGAAGCGGTAG	
17	FBN1F5348	TCAACATGGTTGGCAGCTTCC	476
	FBN1R5824	AAAGATTCCCATTTCCACTTGC	
18	FBN1F5722	ACAATTGGTTCCTTCAACTG	356
	FBN1R6074	GCACAAATTTCTGGCTCTT	
19	FBN1F5973	CTTGGATGGGTCCTACAGATGC	579
	FBN1R6552	CACATTCTTGCAGGTTCCATT	
20	FBN1F6466	GGTTATACTCTAGCGGGAATG	450
	FBN1R6937	TCCCACGGGTGTTGAGGCAGCG	
21	FBN1F6842	AGCGGAGACCTGATGGAGAGG	645
	FBN1R7487	CAGTTGTGTTGCTTGGTTGCA	
22	FBN1F7429	CAAGAGGATGGAAGGAGCTGC	476
	FBN1R7905	GAACTGTTCATACTGGAAGCCG	
23	FBN1F7785	CTACCTCCAGCACTACCAGTGG	384
	FBN1R8169	GTAGCCATTGATCTTACACTCG	
24	FBN1F8024	CACCTGGTTACTTCCGCATAGG	672
	FBN1R8696	ATGATTCTGATTGGGGGAAAA	
